# The Role of Routine Staging Laparoscopy for Pancreatic Adenocarcinoma in Underserved Populations: A 14‐Year Experience at a Safety‐Net Hospital

**DOI:** 10.1002/jso.70299

**Published:** 2026-06-07

**Authors:** David M. Holmes, Jose Euberto Mendez Reyes, Cary Hsu, Eric J. Silberfein

**Affiliations:** ^1^ Department of Surgery Baylor College of Medicine Houston Texas USA

**Keywords:** occult metastases, pancreatic ductal adenocarcinoma, peritoneal washings, positive cytology, safety‐net hospital, staging laparoscopy

## Abstract

**Background:**

There are no consensus guidelines for staging laparoscopy (SL) in pancreatic ductal adenocarcinoma (PDAC) and existing data largely reflect insured referral populations. Routine SL has not been studied in underserved patient populations who face disparities in cancer diagnosis and treatment. We evaluated the utility of SL for potentially operable PDAC at an urban safety‐net hospital and assessed its association with survival.

**Methods:**

A single‐institution retrospective review was performed of all patients undergoing SL for potentially resectable PDAC (May 2011–May 2025). The primary outcome was detection of occult metastasis. Associated factors were assessed using multivariable logistic regression. Survival was analyzed using Kaplan–Meier methods and log‐rank tests.

**Results:**

Fifty‐two patients underwent SL and 18 (35%) received curative‐intent surgery. Median age was 58 years, 87% were non‐White, 40% non‐English‐speaking, and 89% presented emergently. SL identified occult metastatic disease in 12 patients (23.1%), including five (41.7%) identified by cytology alone. No clinicopathologic factors were associated with occult metastasis. Median overall survival was 9.5 months with occult metastasis versus 35.7 months without (*p* < 0.001), and 102.5 months after curative‐intent surgery versus 17.8 months without resection (*p* < 0.001).

**Conclusions:**

Routine SL frequently upstaged patients, often by cytology alone, thereby avoiding non‐therapeutic laparotomy and supporting consideration of its more routine use in underserved PDAC populations.

**Abbreviations**
BTH: Ben Taub Hospital
CT: computed tomography
MRI: magnetic resonance imaging
NAC: Neoadjuvant chemotherapy
NNT: number needed to treat
PDAC: Pancreatic ductal adenocarcinoma
SL: Staging laparoscopy

## 1 Introduction

Pancreatic ductal adenocarcinoma (PDAC) is an aggressive solid tumor malignancy with a poor prognosis. Half of all patients present with metastatic disease and an additional 30%–35% of patients present with locally advanced, unresectable disease [1]. The 5‐year survival remains dismal at less than 15% despite ongoing advances in multimodal treatment strategies [2]. The incidence of pancreas cancer continues to rise in the US and is projected to be the second leading cause of cancer‐related death by 2030 [2, 3].

Surgical resection is central to curative‐intent therapy for PDAC. However, 7%–12% of pancreatic cancer resection attempts are aborted due to unresectable disease or the discovery of occult metastatic disease not identified on preoperative imaging [4, 5]. Staging laparoscopy (SL) plays an important role in the evaluation of PDAC to detect occult metastases and prevent non‐therapeutic laparotomy. Since its introduction in the 1980s, the reported yield of SL has decreased from 30% to 50% in the early 2000s to 15%–25% in the 2010s [6, 7, 8, 9, 10]. These lower detection rates are attributable to significantly improved imaging technology and a shift in the treatment paradigm for locally advanced pancreatic cancers to receive neoadjuvant therapy [11, 12, 13, 14].

There are no consensus criteria guiding the use of SL for PDAC [15]. Certain clinicopathologic factors, such as pancreatic tail lesions and elevated CA 19‐9, are associated with a higher likelihood of detecting radiographically occult metastatic disease, and prognostic scores have been developed to select for high‐risk patients [6]. However, these models do not incorporate social determinants of health including race, socioeconomic and insurance status, and primary language. Prior studies have shown that patients of minority race and lower socioeconomic status are more likely to present with advanced (stage III/IV) disease, less likely to receive adjuvant therapy, and experience worse overall and disease‐specific survival in hepatobiliary malignancies [16, 17, 18, 19, 20]. Despite these disparities, data on the use of SL for PDAC in underserved populations are lacking, and its role in this context remains undefined. We hypothesized that patients from underserved populations are more likely to harbor occult metastatic disease detectable at SL, supporting consideration of its routine use in this group.

The purpose of this study was to evaluate the yield and clinical utility of routine SL in underserved patient populations with potentially operable PDAC at an urban, academic, safety‐net hospital. Specifically, we sought to determine the prevalence of occult metastatic disease and characterize survival according to SL findings.

## 2 Materials and Methods

### 2.1 Study Design and Setting

We conducted a single‐institution retrospective review of all patients referred for staging laparoscopy for potentially operable pancreatic ductal adenocarcinoma (PDAC) between May 2011 and May 2025 at Ben Taub Hospital (BTH), a county‐funded safety‐net hospital located in the Texas Medical Center in Houston, Texas. As a high‐volume academic teaching hospital affiliated with the Baylor College of Medicine, BTH provides care to over one million residents of Harris County who are uninsured, underinsured, or otherwise socioeconomically disadvantaged. The study was approved by the Baylor College of Medicine Institutional Review Board. BTH is a high‐volume center for pancreas surgery, consistently performing greater than 20 pancreas resections annually.

### 2.2 Institutional Treatment Pathway for PDAC

At our institution, staging laparoscopy (SL) for PDAC was performed in patients with histologically confirmed disease who were considered potential candidates for curative resection based on radiographic evaluation and multidisciplinary tumor board consensus. Staging included computed tomography (CT) of the chest, abdomen, and pelvis, with tumor resectability determined by triple‐phase CT scan and/or pancreas‐protocol magnetic resonance imaging (MRI). While the implementation of neoadjuvant therapy evolved during the study period and was not uniform, SL was consistently performed as a separate procedure following histologic diagnosis prior to proceeding with therapeutic surgical resection. No patients in this cohort underwent pancreaticoduodenectomy during the same anesthetic as staging laparoscopy. Patients who initially presented with metastatic disease or had a final pathology that differed from PDAC were excluded.

Staging laparoscopy was performed consistently by three surgeons during the study period. All SLs involved systematic inspection of visible peritoneal surfaces in all four quadrants, the pelvis, the liver surface, greater omentum, and visible small bowel. The lesser sac was not routinely inspected nor was the small bowel run in its entirety. Any identifiable nodules or abnormal appearing tissue were biopsied and sent for histopathologic evaluation. If present, ascites was collected and sent as a separate specimen. Peritoneal lavage was performed by instilling 1 liter of normal saline into the peritoneal cavity and aspirating the fluid for cytologic evaluation. Since 2013, peritoneal lavage has been performed routinely unless gross metastatic disease is identified at the time of staging laparoscopy, in which case a tissue biopsy is performed, and peritoneal lavage is deferred. Prior to 2013, peritoneal lavage was performed at the discretion of the operating surgeon.

### 2.3 Patient and Tumor Characteristics

Data were extracted from the electronic medical record. Patient demographic data analyzed included age, sex, race, alcohol and tobacco use, the presence of diabetes at time of diagnosis, and mode of presentation (emergency department vs. elective referral). Tumor‐specific variables included tumor size, anatomic location, lymph node status, and resectability (resectable, borderline resectable, or locally advanced) as defined by the National Comprehensive Cancer Network (NCCN) guidelines (version 2.2025). Treatment‐related variables included receipt of neoadjuvant chemotherapy, occurrence of laparotomy, reason for aborted resection (if applicable), preoperative CA 19‐9 and CEA levels following bilirubin normalization (if applicable).

### 2.4 Staging Laparoscopy and Outcomes Definitions

The primary outcome was detection of metastatic disease at laparoscopy, defined by the presence of malignant cells on final histopathology of a tissue biopsy and/or cytology from ascites or peritoneal washings. Additional SL variables included biopsies sites, presence or absence of ascites, and performance of peritoneal lavage for cytologic evaluation. Laparotomies were classified as therapeutic if tumor resection was performed and non‐therapeutic if the surgery was aborted due to occult metastatic disease or unresectable, locally advanced disease.

### 2.5 Statistical Analysis

Median follow‐up time and overall survival was determined by vital status at each patient's last documented encounter. Descriptive summary statistics were performed on the study cohort. Median overall survival and recurrence‐free survival was compared using the Kaplan‐Meier method. Univariable and multivariable logistic regression was used to investigate risk factors for detecting metastatic disease at staging laparoscopy. Variables assessed included male gender, borderline resectable and locally advanced disease, CA‐19‐9 level > 394 (U/L), neoadjuvant chemotherapy, tumor location in the body or tail of the pancreas. Number needed to treat (NNT) was calculated to identify number of SLs necessary to avoid one non‐therapeutic laparotomy. Analyses were performed in R. Statistical significance was accepted at a p value of 0.05 or less.

## 3 Results

### 3.1 Patient Characteristics and Treatment Pathway

**FIGURE 1:**
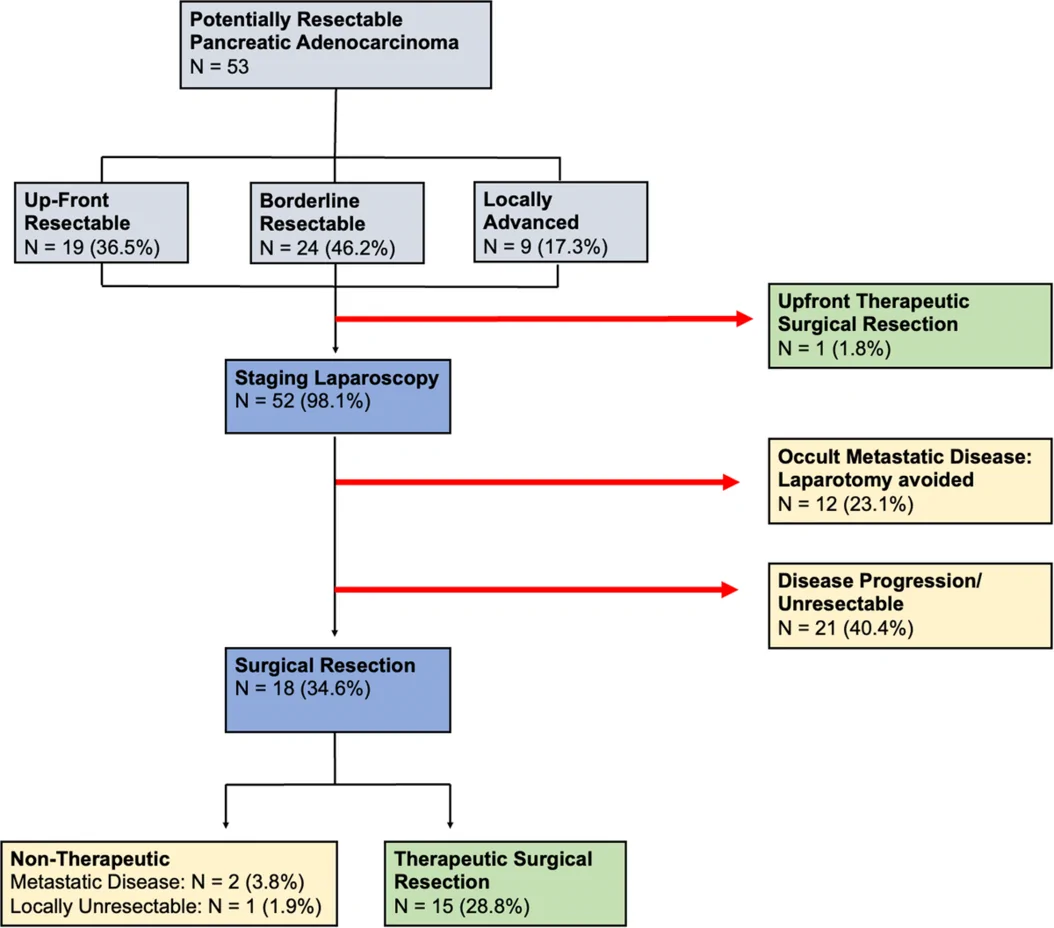
Institutional treatment pathway for the management of potentially operable pancreatic ductal adenocarcinoma.

Between May 2011 and May 2025, 53 patients were referred for surgical treatment of their pancreatic ductal adenocarcinoma (PDAC). Median age was 58 years, 54% were male, 87% were non‐White, 40% were non‐English‐speaking, 52% had diabetes on presentation, 54% reported tobacco use and 31% reported alcohol use, and 89% presented through the emergency department. For those with normal bilirubin, median preoperative CA 19‐9 was 364 U/mL (IQR 69.5–1063.7) and CEA 2.87 ng/mL (IQR 1.46–5.45) (Table 1). Fifty‐two patients (98%) underwent SL and 18 (35%) ultimately received curative‐intent surgery. There was a single patient during the study period with upfront resectable PDAC who proceeded directly to therapeutic resection (Figure 1). Patients who did not proceed to curative‐intent surgery either demonstrated disease progression while on neoadjuvant therapy (NAC) or were ultimately deemed unresectable at multidisciplinary tumor board discussion and placed on definitive chemotherapy. In total, NAC was initiated in 33 patients (63.5%), with 16 (30.7%) initiating NAC prior to the staging laparoscopy. Among these patients, the median interval from initiation of chemotherapy to staging laparoscopy was 125 days (IQR 79–192). At the time of definitive surgery, two patients (3.8%) were found to have occult metastatic disease not previously detected at SL and one had unresectable vascular invasion intraoperatively (Figure 1).

**TABLE 1:** Characteristics of 52 patients who underwent staging laparoscopy for pancreatic ductal adenocarcinoma.

Characteristic	Data (*N* = 52) N (%)
Age, years (IQR)	58 (52–65)
Male Sex	28 (54%)
BMI (Median, IQR)	22.2 (19.4–26.2)
CA 19‐9, U/mL (IQR)	364 (69.5–1063.7)
Race/Ethnicity:	
Hispanic	22 (42.3)
Black	17 (32.7)
White	7 (13.5)
Other Minority	6 (11.5)
Social/Medical Factors:	
Tobacco use	28 (53.8)
Alcohol use	16 (30.8)
Diabetes	27 (51.9)
English as Primary Language	31 (59.6)
Mode of Presentation	
Emergent	46 (88.5)
Elective Referral	6 (11.5)
Tumor Size, cm (IQR)	3.08 (2.3–3.7)
Tumor Location	
Head/Uncinate	44 (84.6)
Body/Tail	8 (15.4)
Tumor Resectability	
Up Front Resectable	19 (36.5)
Borderline Resectable	24 (46.2)
Locally Advanced	9 (17.3)

### 3.2 Tumor Characteristics

Forty‐four patients (84.6%) presented with tumors located in the pancreatic head or uncinate process and eight (15.4%) in the body or tail. Median tumor size was 3.1 cm (IQR 2.3–3.7). By radiographic criteria, 19 (36.5%) were classified as resectable, 24 (46.2%) as borderline resectable, and 9 (17.3%) as locally advanced (Table 1). Thirty‐seven patients (71.1%) demonstrated macrovascular invasion of either the common hepatic artery, superior mesenteric artery or vein, portal vein, or inferior vena cava. Twenty‐one patients (41%) had lymphadenopathy present on CT or MRI imaging.

### 3.3 Staging Laparoscopy Outcomes and Number Needed to Treat

SL identified occult metastatic disease in 12 patients (23.1%, Table 2). Ascites was present in five patients (9.4%), of whom two had final cytology positive for adenocarcinoma. Tissue biopsies were performed in 21 patients (40.4%), most commonly from the liver (50%), non‐diaphragmatic peritoneal surfaces (27%), and diaphragm (19%). Peritoneal lavage for cytology was performed in 35 patients (67.3%), of whom 7 (20.6%) demonstrated adenocarcinoma. Among patients with occult metastatic disease, five (42%) were diagnosed exclusively by positive cytology in the absence of gross metastatic disease (Table 2). Although positive SLs occurred more frequently in borderline resectable or locally advanced disease than in upfront resectable disease (10/33 patients vs. 2/19 patients), this difference was not statistically significant (Table 2). Logistic regression did not demonstrate any factors associated with metastatic disease (Table 3). All patients found to have occult metastatic disease were placed on palliative chemotherapy with subsequent disease progression and death.

**TABLE 2:** Staging laparoscopy results according to tumor resectability on CT.

Tumor	Number	Positive Laparoscopy	Macroscopic Metastasis	Positive Cytology Only
Upfront Resectable	19	2	1	1
Borderline Resectable	24	8	4	4
Locally Advanced	9	2	2	0
Total	52	12	7	5

**TABLE 3:** Multivariable logistic regression analysis of risk factors for detecting occult metastatic disease.

Variable	OR	95% CI	*p*‐value
Male gender	1.62	0.40–7.14	0.501
Resectability: Borderline	5.54	0.86–52.10	0.091
Resectability: Locally advanced	2.09	0.19–23.43	0.526
CA 19‐9 > 394 U/L	1.74	0.41–7.75	0.451
Tumor location: Body/Tail	2.15	0.21–18.66	0.482

The absolute risk reduction for SL in preventing non‐therapeutic laparotomy was 17.4%, as 23.1% of patients were upstaged while 5.7% still underwent non‐therapeutic laparotomy despite a negative SL. Accordingly, the number needed to treat (NNT) to prevent one non‐therapeutic laparotomy was six SLs.

### 3.4 Survival Analysis

**FIGURE 2:**
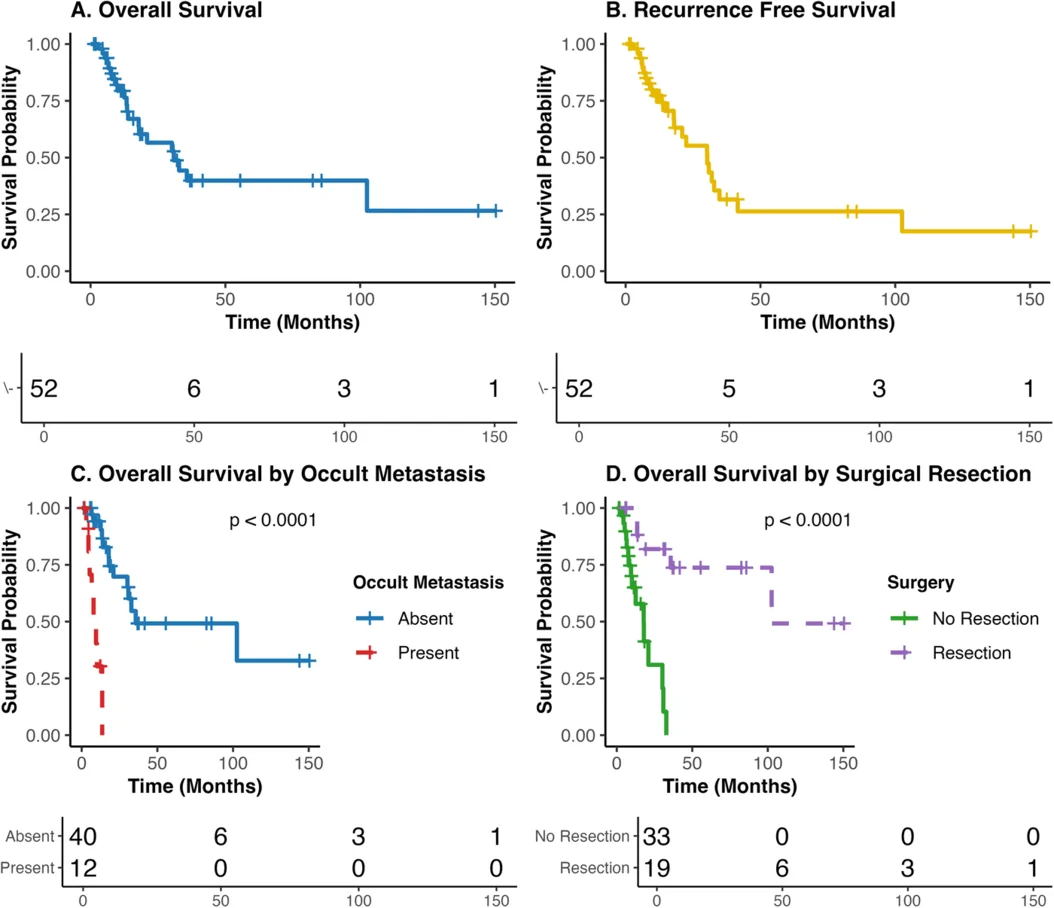
Kaplan‐Meier estimates of overall and recurrence‐free survival, including subgroup analyses by occult metastasis and surgical resection.

Median follow‐up time was 30.8 months. Median overall survival for the cohort was 30.8 months, and median recurrence‐free survival was 30.3 months. Survival differed markedly by group, with a median survival of 35.7 months in patients without occult metastasis versus 9.5 months with occult metastasis (log‐rank *p* < 0.001). Patients who proceeded to curative‐intent surgery had the longest median survival time of 102.5 months versus 17.8 months without resection (log‐rank *p* < 0.001) (Figure 2).

## 4 Discussion

In this study, we report a 14‐year experience of treating pancreatic ductal adenocarcinoma (PDAC) at a major academic, urban safety‐net hospital in Houston, Texas. We sought to determine the utility of routine staging laparoscopy (SL) in our vulnerable patient population who face disparities in cancer diagnosis and treatment leading to overall lower survival rates [16, 17, 18, 19]. We hypothesized that this high‐risk patient population, who often present with late‐stage disease, would be more likely to have occult metastatic disease detected at SL, warranting its routine use. In total, we found that 12 of the 52 patients (23.1%) who underwent staging laparoscopy for PDAC since 2011 had occult metastatic disease. These patients did not receive curative‐intent surgery, thereby avoiding the morbidity associated with a non‐therapeutic laparotomy. Accordingly, the NNT for staging laparoscopy to avoid one laparotomy was six.

Our occult metastatic detection rate is comparable with several recent studies from the past decade conducted at high‐resource, tertiary, academic referral centers. In 2022, Jambor et al. found that SL led to upstaging in 15.4% of 155 cases with a NNT of eight [10]. Takadate et al. found 33% of 146 patients had occult metastases in a Japanese cohort [21]. Similarly, Fong et al. evaluated 1001 patients from 2001 to 2015 and found that SL avoided unnecessary laparotomy in 44.4% of patients between 2001 and 2008 and in 24.1% between 2009 and 2015. Collectively, these contemporary detection rates represent marked improvements compared with the initial experience reported by Warshaw et al. in 1986, when occult metastatic disease was identified in 35% of cases [22], likely reflecting advances in preoperative imaging, laparoscopic technology, and the increasing use of neoadjuvant therapy.

Although there is no consensus regarding which patients should receive staging laparoscopy, the NCCN guidelines (Version 2.2025) recommend its selective use in patients with PDAC who are considered high‐risk for occult metastatic disease [23]. These include patients with borderline resectable or locally advanced lesions, those with CA 19‐9 levels higher than 100 U/L, and those with pancreatic body or tail lesions. Fong et al. developed a scoring system using these factors to stratify their patients into high or low‐risk groups [6]. In our study, positive SLs occurred more frequently in patients with borderline resectable or locally advanced disease compared with those with upfront resectable tumors (10/33 vs. 2/19), although this difference was not statistically significant. No other factors were associated with occult metastasis on logistic regression, likely reflecting the limited sample size and statistical power of our cohort rather than absence of true association. Notably, staging laparoscopy was performed in a near‐universal fashion during the study period. While this limits the ability to perform comparisons with a non‐staging cohort, it minimizes selection bias and allows for evaluation of its diagnostic yield in an unselected, real‐world population of underserved patients within a safety‐net setting, rather than a pre‐selected subgroup based on traditional high‐risk clinicopathologic features or scoring systems. In this context, our findings support the hypothesis that underserved populations may represent a distinct high‐risk group for radiographically occult metastatic disease not fully captured by existing selection criteria. Because current algorithms rely predominantly on tumor‐specific factors, they may underestimate metastatic risk in populations where delayed presentation and more advanced disease burden at diagnosis are more common than in the general population.

The optimal timing of SL also remains controversial, specifically whether it should be done as a separate procedure or at the time of curative‐intent surgery. At our institution, all patients evaluated for potentially resectable PDAC routinely underwent SL as a separate procedure prior to surgical resection, allowing for peritoneal lavage and analysis of cytology. There are a limited number of studies evaluating the role of diagnostic laparoscopy immediately prior to curative‐intent surgery. Chang et al. (2024) found that only three of 126 patients (2.4%) who received diagnostic laparoscopy prior to resection had gross metastatic disease requiring termination of the surgery (NNT = 42). The authors acknowledged that a major limitation of same‐procedure diagnostic laparoscopy is the inability to evaluate peritoneal cytology [24]. In our study, we found that among patients with occult metastatic disease, two‐thirds had positive cytology by peritoneal washings or ascites (eight of 12) and 42% (five of 12) were diagnosed exclusively by cytology in the absence of gross metastatic disease, underscoring the importance of routine peritoneal washings at staging laparoscopy. The Jambor et al. study reinforces this finding, with 45.8% of their patient cohort diagnosed with occult metastatic disease on cytology alone [10].

The NCCN guidelines consider positive cytology from washings obtained at laparoscopy to be equivalent to M1 disease. As such, all patients at our institution who had occult metastatic disease were placed on definitive chemotherapy with subsequent disease progression and death (Figure 1). However, the optimal management of patients who convert from positive to negative cytology after neoadjuvant chemotherapy remains poorly defined. Several studies demonstrate inferior outcomes for patients who undergo surgical resection with positive cytology compared with those with negative cytology. Ferrone et al. reported a median survival of 8 months for resected patients with positive cytology versus 16 months for those with negative cytology [24], while Aoki et al. found significantly improved survival among patients who converted to cytology‐negative after neoadjuvant therapy compared with those who remained positive (30.8 vs. 14.8 months) [25]. Similarly, Jambor et al. reported comparable survival for patients resected after negative SL and those with initially positive SL who ultimately underwent resection (34 vs. 32 months). Both groups had markedly better survival than patients with positive SL who were not resected (6 months; *p* = 0.003). In our cohort, the discovery of any occult metastasis had a significant impact on overall survival with a median overall survival of 35.7 months in patients without occult metastasis versus 9.5 months with occult metastasis (*p* < 0.001). Patients who were able to proceed to curative‐intent surgery had the longest median survival of 102.5 months versus 17.8 months without resection (*p* < 0.001). While further prospective studies are needed to better define the role of resection in patients who convert to negative cytology after neoadjuvant therapy, our findings reinforce the prognostic importance of staging laparoscopy for underserved PDAC populations and support its role in guiding surgical decision‐making.

This study had several limitations. First, this was a retrospective, single‐center study, with a small patient cohort with limited statistical power. Second, our median follow‐up time was limited due to ongoing data collection until May 2025. Additionally, our institution's protocol for the administration of neoadjuvant chemotherapy for PDAC evolved during the study period, and timing of NAC relative to staging laparoscopy was not standardized. A total of 16 patients (30.7%) received NAC prior to staging laparoscopy, which may have impacted the occult metastatic detection rate. Staging laparoscopy technique was also not standardized among surgeons, and peritoneal cytology was not routinely performed during the entire study period, which may have led to under‐detection of occult metastatic disease. Finally, although our cohort included all patients undergoing attempted curative‐intent resection for PDAC during the study period, only one patient proceeded directly to surgery without staging laparoscopy. Therefore, the absence of a meaningful control group precluded comparative outcome analyses.

## 5 Conclusions

Staging laparoscopy is a valuable tool in the management of potentially operable pancreatic ductal adenocarcinoma in high‐risk safety‐net settings, aiding operative decision‐making and promoting more equitable oncologic care. At our institution, approximately one in four patients undergoing SL was upstaged to metastatic disease, thereby preventing non‐therapeutic laparotomy and supporting consideration of its more routine use in underserved PDAC populations.

## Synopsis

This study evaluated the yield and clinical utility of routine staging laparoscopy for pancreatic ductal adenocarcinoma in an underserved patient population over a 14‐year period. We found that staging laparoscopy identified occult metastatic disease in nearly one in four patients, altering management and preventing non‐therapeutic laparotomy. These findings support routine staging laparoscopy as a valuable tool to improve operative decision‐making and promote equitable oncologic care in high‐risk safety‐net settings.

## Data Availability

The data that support the findings of this study are available on request from the corresponding author. The data are not publicly available due to privacy or ethical restrictions.
